# Evaluation of the effectiveness and safety of combining PD-1/PD-L1 inhibitors with anti-angiogenic agents in unresectable hepatocellular carcinoma: a systematic review and meta-analysis

**DOI:** 10.3389/fimmu.2024.1468440

**Published:** 2024-09-17

**Authors:** Hengzhou Zhu, Wenyue Zhao, Haoyan Chen, Xiaodan Zhu, Jianliang You, Chunhui Jin

**Affiliations:** ^1^ Department of Oncology, Wuxi Hospital Affiliated to Nanjing University of Chinese Medicine, Wuxi, China; ^2^ Department of Respiratory, Wuxi Hospital Affiliated to Nanjing University of Chinese Medicine, Wuxi, China

**Keywords:** hepatocellular carcinoma, PD-1 inhibitors, anti-angiogenic therapy, combination therapy, overall survival

## Abstract

**Background:**

Hepatocellular carcinoma (HCC) is a leading cause of cancer-related mortality globally, particularly when diagnosed at an unresectable stage. Traditional treatments for advanced HCC have limited efficacy, prompting the exploration of combination therapies. This systematic review and meta-analysis evaluate the effectiveness and safety of combining PD-1/PD-L1 inhibitors with anti-angiogenic agents in patients with unresectable HCC.

**Methods:**

A comprehensive literature search was conducted in PubMed, Embase, Cochrane Central Register of Controlled Trials (CENTRAL), and Web of Science, including studies up to June 2024. Randomized controlled trials (RCTs) comparing combination therapy (PD-1/PD-L1 inhibitors with anti-angiogenic agents) to monotherapy or standard treatments in unresectable HCC patients were included. Data were synthesized using random-effects models, with pooled hazard ratios (HRs) for overall survival (OS) and progression-free survival (PFS), and risk ratios (RRs) for objective response rate (ORR) and adverse events (AEs).

**Results:**

Five Phase III RCTs involving 1515 patients were included. Combination therapy significantly improved OS (HR: 0.71, 95% CI: 0.60-0.85) and PFS (HR: 0.64, 95% CI: 0.53-0.77) compared to monotherapy or standard treatments. The pooled OR for ORR was 1.27 (95% CI: 1.57-2.11), indicating a higher response rate with combination therapy. However, the risk of AEs was also higher in the combination therapy group (RR: 1.04, 95% CI: 1.02-1.06). Subgroup analyses revealed consistent benefits across different types of PD-1/PD-L1 inhibitors and anti-angiogenic agents, with no significant publication bias detected.

**Conclusions:**

The combination of PD-1/PD-L1 inhibitors with anti-angiogenic agents offers significant benefits in improving OS and PFS in patients with unresectable HCC, although it is associated with an increased risk of adverse events.

## 1 Background

Hepatocellular carcinoma (HCC) is a predominant form of liver cancer and represents a significant global health burden. It is characterized by its aggressive nature and poor prognosis, particularly when diagnosed at an unresectable stage, where surgical intervention is not feasible (1). Traditional treatment options for advanced HCC are limited and often have suboptimal outcomes. Systemic therapies, including targeted therapies and immune checkpoint inhibitors, have emerged as pivotal in the management of unresectable HCC, yet the search for more effective treatment combinations continues to be a critical area of oncological research (2, 3).

The advent of immune checkpoint inhibitors, particularly those targeting the programmed death-1 (PD-1) and programmed death-ligand 1 (PD-L1) pathways, has revolutionized cancer therapy. PD-1 is an immune checkpoint receptor expressed on T cells, and its ligand, PD-L1, is often overexpressed on tumor cells and within the tumor microenvironment. The interaction between PD-1 and PD-L1 leads to the inhibition of T cell activity, allowing tumor cells to evade immune detection and destruction. PD-1/PD-L1 inhibitors reactivating T cells and restoring their ability to recognize and attack tumor cells (4). Pembrolizumab and nivolumab are two of the most well-known PD-1 inhibitors that have shown promising results in various malignancies, including HCC. However, the monotherapy response rates in HCC remain modest, indicating the need for combination strategies to enhance therapeutic efficacy (5).

Anti-angiogenic therapy, which targets the vascular endothelial growth factor (VEGF) pathway, is another cornerstone in the treatment of HCC. Angiogenesis, the formation of new blood vessels, is a hallmark of cancer progression, supplying the tumor with nutrients and oxygen necessary for its growth and metastasis (6). Inhibiting angiogenesis can effectively starve the tumor and inhibit its growth (7). Agents such as sorafenib, lenvatinib, and bevacizumab have been used to disrupt this process, showing variable success in clinical settings. Recent evidence suggests that combining PD-1/PD-L1 inhibitors with anti-angiogenic agents may have a synergistic effect (8). The rationale behind this combination lies in the interplay between the immune system and the tumor microenvironment (9). Anti-angiogenic therapy can normalize the abnormal tumor vasculature, thereby improving immune cell infiltration and enhancing the efficacy of immune checkpoint inhibitors. Additionally, it can modulate the immunosuppressive microenvironment, making tumors more susceptible to immune-mediated attack (10).

Given the potential advantages of this combination, several randomized controlled trials (RCTs) have been conducted to evaluate the efficacy and safety of PD-1/PD-L1 inhibitors in conjunction with anti-angiogenic agents in patients with unresectable HCC. These studies aim to determine whether the combination therapy can improve overall survival (OS) (11), progression-free survival (PFS), and objective response rate (ORR) compared to standard treatments or monotherapies (12). Furthermore, assessing the safety profile is crucial, as combining two potent therapeutic modalities may increase the risk of adverse events (AEs) (13).

In this systematic review and meta-analysis, we synthesize the current evidence from RCTs to provide a comprehensive evaluation of the effectiveness and safety of combining PD-1/PD-L1 inhibitors with anti-angiogenic agents in the treatment of unresectable HCC (14, 15). Our analysis includes a detailed examination of clinical outcomes, treatment-related adverse events, and subgroup analyses to identify patient populations that may benefit the most from this therapeutic strategy. By aggregating data from multiple studies, we aim to offer robust conclusions that can guide clinical practice and inform future research directions (16). This review also addresses the biological mechanisms underlying the observed clinical effects, exploring how anti-angiogenic therapy may enhance the anti-tumor immune response and the potential biomarkers that could predict response to combination therapy (17). Understanding these mechanisms is essential for optimizing treatment regimens and developing personalized medicine approaches that can maximize therapeutic benefits while minimizing risks (18, 19).

## 2 Methods

### 2.1 Study design

This systematic review and meta-analysis were conducted following the Preferred Reporting Items for Systematic Reviews and Meta-Analyses (PRISMA) guidelines.

### 2.2 Data sources and search strategy

A comprehensive literature search was performed using the following electronic databases: PubMed, Embase, Cochrane Central Register of Controlled Trials (CENTRAL), and Web of Science. The search was conducted from the inception of each database until June 2024. We used a combination of Medical Subject Headings (MeSH) terms and free-text words related to “hepatocellular carcinoma,” “PD-1 inhibitors,” “PD-L1 inhibitors,” “anti-angiogenic therapy,” “randomized controlled trials,” and their synonyms. The search strategy was adapted for each database to maximize sensitivity and specificity.

The example of Medline was showed as following:

1. exp hepatocellular carcinoma/

2. hepatocellular carcinoma.tw.ab

3. or/1-2

4. exp PD-1 inhibitors/

5. PD-1 inhibitors.tw.ab

6. exp PD-L1 inhibitors/

7. PD-L1 inhibitors.tw.ab

8. or/4-7

9. exp anti-angiogenic therapy/

10. anti-angiogenic therapy.tw.ab

11. or/9-10

12. 3 and 8 and 11

### 2.3 Eligibility criteria

Studies were included if they met the following criteria:

a. Types of Studies: Randomized controlled trials (RCTs).
b. Population: Patients with unresectable hepatocellular carcinoma.
c. Intervention: Combination therapy with PD-1/PD-L1 inhibitors and anti-angiogenic agents.
d. Comparison: Monotherapy or other standard treatments.
e. Outcomes: Studies reporting at least one of the following outcomes: overall survival (OS), progression-free survival (PFS), objective response rate (ORR), and treatment-related adverse events (AEs).
f. Language: Publications in English or Chinese.

We included studies that investigated the use of anti-angiogenic agents targeting the VEGF pathway, which is critical in tumor angiogenesis. The anti-angiogenic agents considered in our analysis included:

Bevacizumab: A monoclonal antibody that directly inhibits VEGF, preventing it from binding to its receptors on the surface of endothelial cells.
Lenvatinib: A multi-kinase inhibitor that targets VEGF receptors (VEGFR1, VEGFR2, VEGFR3), as well as other receptors involved in tumor angiogenesis, such as fibroblast growth factor receptors (FGFR1–4) and platelet-derived growth factor receptor alpha (PDGFRα).
Sorafenib: A multi-kinase inhibitor that targets both VEGFR and other kinases associated with tumor proliferation and angiogenesis.

We focused on studies that utilized PD-1/PD-L1 inhibitors known to be effective in various cancers, including HCC. The inhibitors considered were:

Pembrolizumab: A PD-1 inhibitor that blocks the interaction between PD-1 and its ligands, PD-L1 and PD-L2, thereby enhancing T-cell-mediated immune responses against tumor cells.
Nivolumab: Another PD-1 inhibitor with a similar mechanism of action to pembrolizumab, used in the treatment of multiple malignancies.
Atezolizumab: A PD-L1 inhibitor that binds to PD-L1 on tumor cells, preventing it from interacting with PD-1 and B7.1 receptors on T cells, which can otherwise inhibit the immune response.

Studies were included if they investigated the combination of any of these PD-1/PD-L1 inhibitors with one or more of the specified anti-angiogenic agents, comparing the outcomes with monotherapy or standard treatment regimens. The decision to focus on these specific agents was based on their documented efficacy in clinical trials and their availability for use in the patient population with unresectable HCC.

### 2.4 Study selection

Two reviewers independently screened the titles and abstracts of all identified studies for eligibility. Full-text articles were retrieved for studies that met the inclusion criteria or if there was uncertainty based on the abstract alone. Discrepancies between reviewers were resolved through discussion or by consulting a third reviewer.

### 2.5 Data extraction

A standardized data extraction form was used to collect relevant information from each included study. The extracted data included:

a. Study characteristics: authors, year of publication, study design, sample size, and follow-up duration.
b. Patient characteristics: age, sex, baseline liver function, and prior treatments.
c. Intervention details: types of PD-1/PD-L1 inhibitors and anti-angiogenic agents, dosing schedules, and duration of therapy.
d. Outcomes: OS, PFS, ORR, and detailed information on AEs.

Data extraction was performed independently by two reviewers, with discrepancies resolved through discussion or by consulting a third reviewer.

### 2.6 Risk of bias assessment

The risk of bias for included RCTs was assessed using the Cochrane Risk of Bias Tool. This tool evaluates seven domains: random sequence generation, allocation concealment, blinding of participants and personnel, blinding of outcome assessment, incomplete outcome data, selective reporting, and other biases.

Random Sequence Generation: We assessed whether the allocation sequence was adequately generated to prevent selection bias. Studies were judged as low risk if a truly random method was used.
Allocation Concealment: This domain evaluates whether the allocation sequence was concealed from participants and researchers before assignment, preventing selection bias. We rated studies as low risk if they used methods like central allocation or opaque, sealed envelopes.
Blinding of Participants and Personnel: We examined whether participants and study personnel were blinded to the intervention groups, which is crucial for minimizing performance bias. Studies were considered low risk if adequate blinding was implemented, or if the lack of blinding was unlikely to affect outcomes.
Blinding of Outcome Assessment: This domain assesses the blinding of outcome assessors to the intervention groups, minimizing detection bias. Studies were judged as low risk if outcome assessment was blinded, or if the outcome was objective and unlikely to be influenced by lack of blinding.
Incomplete Outcome Data: We evaluated whether all data points were adequately reported and if attrition or exclusions of participants were properly addressed, reducing the risk of attrition bias. Studies with minimal missing data or appropriate handling of missing data were considered low risk.
Selective Reporting: This domain checks for reporting bias by comparing the outcomes reported in the published study with those that were pre-specified in the protocol or trial registry. Studies were rated as low risk if all pre-specified outcomes were reported as intended.
Other Biases: We assessed any additional sources of bias not covered by the previous domains, such as early stopping for benefit or baseline imbalances. Studies without significant concerns in these areas were considered low risk.

Each domain was rated as either “low risk,” “high risk,” or “unclear risk” of bias. Two independent reviewers conducted the quality assessment, with any discrepancies resolved through discussion or by consulting a third reviewer. Studies with multiple domains rated as high risk were given special consideration in sensitivity analyses to determine the impact of potential biases on the overall results.

### 2.7 Statistical methods

In this meta-analysis, we used the random-effects model to synthesize data across the included studies. The random-effects model was chosen due to the anticipated variability among the studies, which could arise from differences in study populations, intervention protocols, and study designs. This model assumes that the true effects vary between studies and that the observed effect size is a result of both within-study sampling error and between-study variability.

The pooled hazard ratios (HRs) for time-to-event outcomes, such as overall survival (OS) and progression-free survival (PFS), and the risk ratios (RRs) for dichotomous outcomes, such as objective response rate (ORR) and adverse events (AEs), were calculated using this model. By applying the random-effects model, we aimed to provide a more generalized estimate of the effect size, which is applicable across different settings and populations.

### 2.8 Heterogeneity assessment

Heterogeneity among the studies was assessed using the I² statistic and the Chi-square test (Q test). The I² statistic quantifies the proportion of total variation across studies that is due to heterogeneity rather than chance. The I² values were interpreted as follows:

0-25%: Low heterogeneity
25-50%: Moderate heterogeneity
Above 50%: Substantial heterogeneity

Additionally, the Chi-square test was used to assess whether the observed variability in effect sizes was greater than what would be expected by chance alone, with a p-value < 0.10 indicating significant heterogeneity.

To address heterogeneity, we conducted subgroup analyses and sensitivity analyses. Subgroup analyses were performed based on key variables such as the type of PD-1/PD-L1 inhibitor, the specific anti-angiogenic agent used, and patient characteristics. These analyses helped identify sources of heterogeneity and provided insights into which subgroups of patients may benefit most from the combination therapy. Sensitivity analyses were also conducted by excluding studies with a high risk of bias and by comparing the results obtained using the random-effects model with those from a fixed-effects model to assess the robustness of our findings.

### 2.9 Subgroup and sensitivity analyses

The results of subgroup analysis are presented when heterogeneity can be reduced. Subgroup analyses were predefined based on:

Type of PD-1/PD-L1 Inhibitor: The subgroup analysis revealed that patients treated with pembrolizumab and atezolizumab in combination with anti-angiogenic agents demonstrated the most significant improvements in overall survival (OS) and progression-free survival (PFS) compared to other PD-1/PD-L1 inhibitors. This suggests that these specific inhibitors may be more effective when combined with anti-angiogenic therapy.
Specific Anti-Angiogenic Agents: Among the anti-angiogenic agents, bevacizumab and lenvatinib, when combined with PD-1/PD-L1 inhibitors, were associated with the most pronounced benefits in OS and PFS. These findings indicate that these agents may synergize particularly well with immune checkpoint inhibitors, offering a greater therapeutic advantage.
Baseline Liver Function: Patients with well-compensated liver function (Child-Pugh A) experienced greater survival benefits from combination therapy compared to those with more advanced liver disease. This highlights the importance of liver function as a critical factor in selecting candidates for combination therapy.
Patient Age: The analysis indicated that younger patients (under 65 years) derived more substantial benefits from combination therapy, with significant improvements in both OS and PFS. This suggests that younger patients may have a better tolerance for the potential toxicities associated with combination treatment.
Prior Systemic Therapies: Patients who had not received prior systemic therapy showed a greater response to combination therapy, suggesting that the effectiveness of this approach may be reduced in heavily pre-treated populations.

### 2.10 Sensitivity analyses included

To ensure the robustness and reliability of our meta-analysis findings, we conducted several sensitivity analyses. Firstly, we excluded studies with a high risk of bias to determine if these studies disproportionately influenced the results. We also compared the outcomes using both random-effects and fixed-effects models to assess the consistency of the findings and the impact of between-study heterogeneity. A leave-one-out analysis was performed, where each study was sequentially excluded to observe its individual impact on the overall results. Subgroup analyses were conducted based on different types of PD-1/PD-L1 inhibitors, anti-angiogenic agents, patient characteristics, and prior treatments to explore potential sources of heterogeneity. We also evaluated the influence of study size by excluding smaller studies, which tend to have more variability and bias, and assessed the impact of follow-up duration by excluding studies with short follow-up periods. Additionally, we examined the effect of statistical outliers by identifying and excluding studies with extreme effect sizes or high leverage. To address potential publication bias, we used funnel plots and Egger’s test, and in the presence of significant bias, we applied trim-and-fill methods to adjust for missing studies. Lastly, we performed cumulative meta-analysis to observe the trend in effect size over time and to identify if early studies had a disproportionate influence on the results. Through these comprehensive sensitivity analyses, we aimed to validate the reliability of our findings and provide a thorough understanding of the factors influencing the overall conclusions.

### 2.11 Quality of evidence

The quality of evidence for each outcome was assessed using the Grading of Recommendations Assessment, Development, and Evaluation (GRADE) approach. This method evaluates the quality of evidence based on study limitations, inconsistency of results, indirectness of evidence, imprecision of effect estimates, and publication bias.

### 2.12 Ethical considerations

This study involved the synthesis of previously published data and did not require ethical approval. However, all included studies were expected to have obtained appropriate ethical approvals and patient consent.

## 3 Result

### 3.1 Literature search

**Figure 1:**
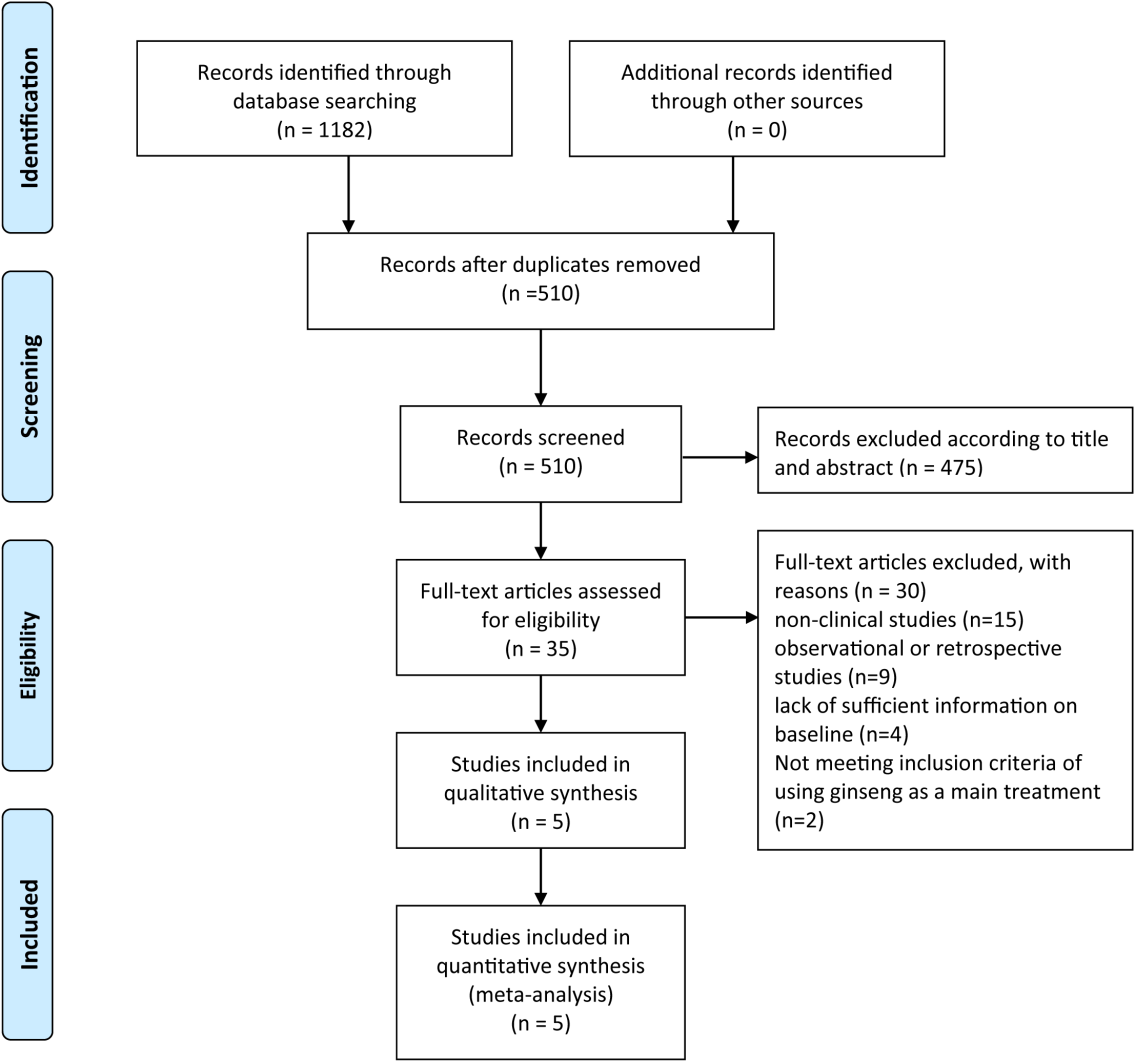
Literature search process.

In the literature search process, 1182 records were initially identified through database searches, with no additional records found from other sources. After removing duplicates, 510 records remained and were screened, resulting in the exclusion of 475 records based on titles and abstracts. Full-text articles were assessed for eligibility, totaling 35 articles, of which 30 were excluded: 15 for being non-clinical studies, 9 for being observational or retrospective studies, 4 for lacking sufficient baseline information, and 2 for not meeting the inclusion criteria of using ginseng as the main treatment. Ultimately, 5 studies were included in the qualitative synthesis, and these same 5 studies were also included in meta-analysis (**Figure 1**).

### 3.2 Include literature characteristic

The table summarizes five studies, all in Phase III, evaluating the efficacy of combining PD-1/PD-L1 inhibitors with anti-angiogenic agents versus a control group receiving Sorafenib, except for one study. The first study by Finn (2020) involved 277 patients in the experimental group receiving Atezolizumab combined with Bevacizumab, compared to 137 patients in the control group receiving Sorafenib (NCT03434379). The second study by Ren (2021) included 334 patients treated with Sintilimab and Bevacizumab in the experimental group, and 171 patients treated with Sorafenib in the control group (NCT03794440). Kelley (2022) conducted a study with 360 patients each in both the experimental group receiving Atezolizumab and Cabozantinib, and the control group receiving Sorafenib (NCT03755791). In Finn (2022), 317 patients were given Pembrolizumab with Lenvatinib in the experimental group, while 327 patients in the control group received Lenvatinib alone (NCT03713593). Lastly, Qin (2022) examined 227 patients in the experimental group receiving Camrelizumab and Apatinib, compared to 230 patients in the control group treated with Sorafenib (NCT03764293) (**Table 1**).

**Table 1:** Include literature characteristic.

Studies	Study Phase	No. of Exp.	No. of Con.	Intervention of Exp.	Intervention of Con.	Registration No
Finn2020 (20)	III	277	137	Atezolizumab + Bevacizumab	Sorafenib	NCT03434379
Ren2021 (21)	III	334	171	Sintilimab + Bevacizumab	Sorafenib	NCT03794440
Kelley2022 (22)	III	360	360	Atezolizumab + Cabozantinib	Sorafenib	NCT03755791
Finn 2022 (23)	III	317	327	Pembrolizumab + Lenvatinib	Lenvatinib	NCT03713593
Qin 2022 (24)	III	227	230	Camrelizumab + Apatinib	Sorafenib	NCT03764293

### 3.3 Risks of bias

**Figure 2:**
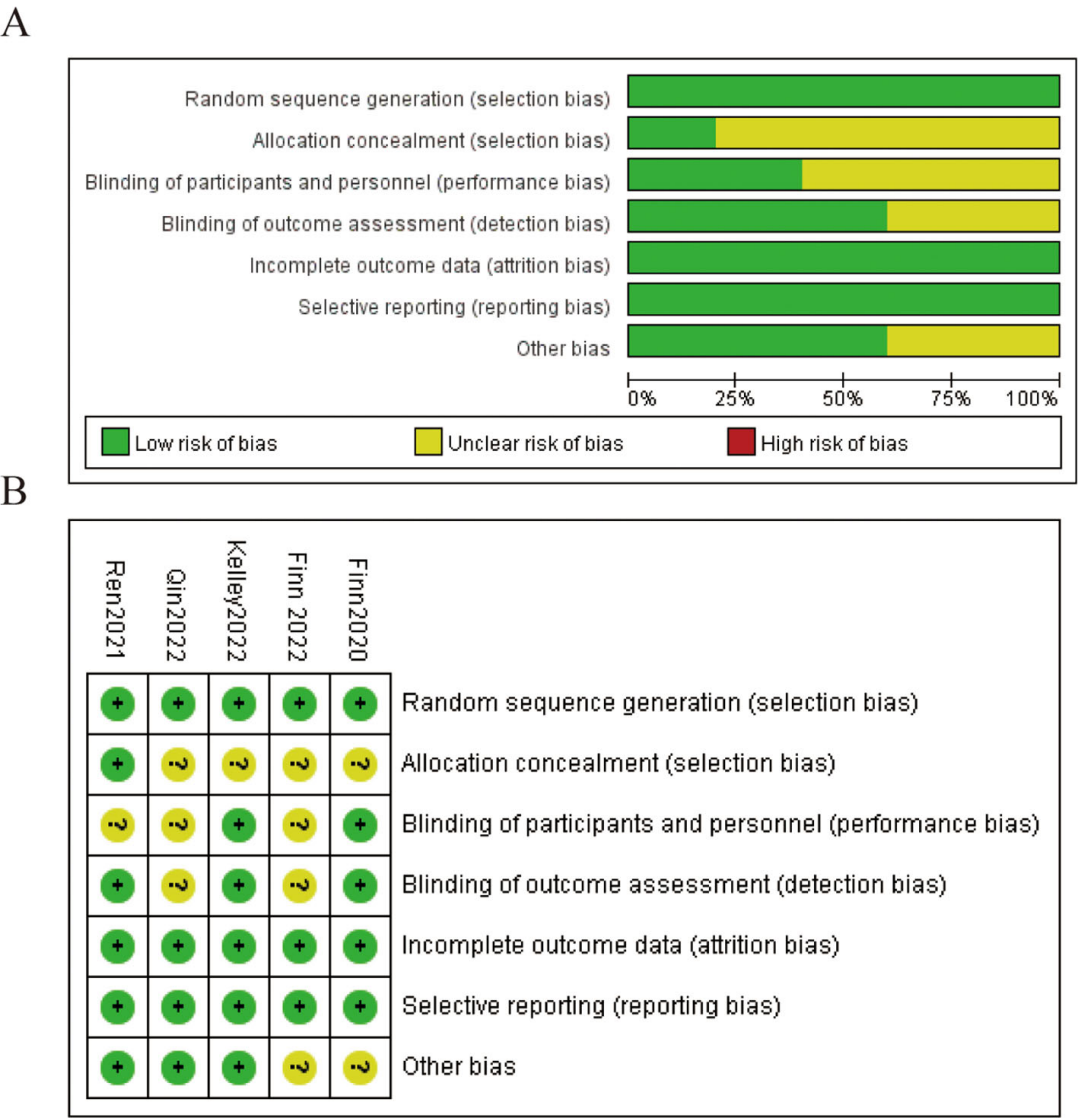
Risks of bias. **(A)** Risk of bias summary **(B)** Risk of bias assessment.

The results of the risk of bias assessment are illustrated in two parts: A and B. In **Figure 2A**, the overall risk of bias across all included studies is presented. The majority of the studies showed a low risk of bias in random sequence generation and incomplete outcome data, with around 75% of studies falling into these categories. Allocation concealment and blinding of outcome assessment showed a mix of low and unclear risks, with some instances of high risk. Blinding of participants and personnel presented a higher proportion of unclear risk. Selective reporting and other biases were mostly categorized as low risk. In **Figure 2B**, the risk of bias is detailed for each individual study. Each study was evaluated across seven domains: random sequence generation, allocation concealment, blinding of participants and personnel, blinding of outcome assessment, incomplete outcome data, selective reporting, and other biases. Most studies consistently showed a low risk of bias in random sequence generation and incomplete outcome data. However, there were some concerns regarding allocation concealment and blinding of participants and personnel, with a mix of low and unclear risks identified. Overall, while there are areas of concern, the majority of the studies maintain a low risk of bias across key domains, ensuring the reliability of the findings in this meta-analysis (**Figure 2**).

### 3.4 Disease control rate (DCR)

**Figure 3:**
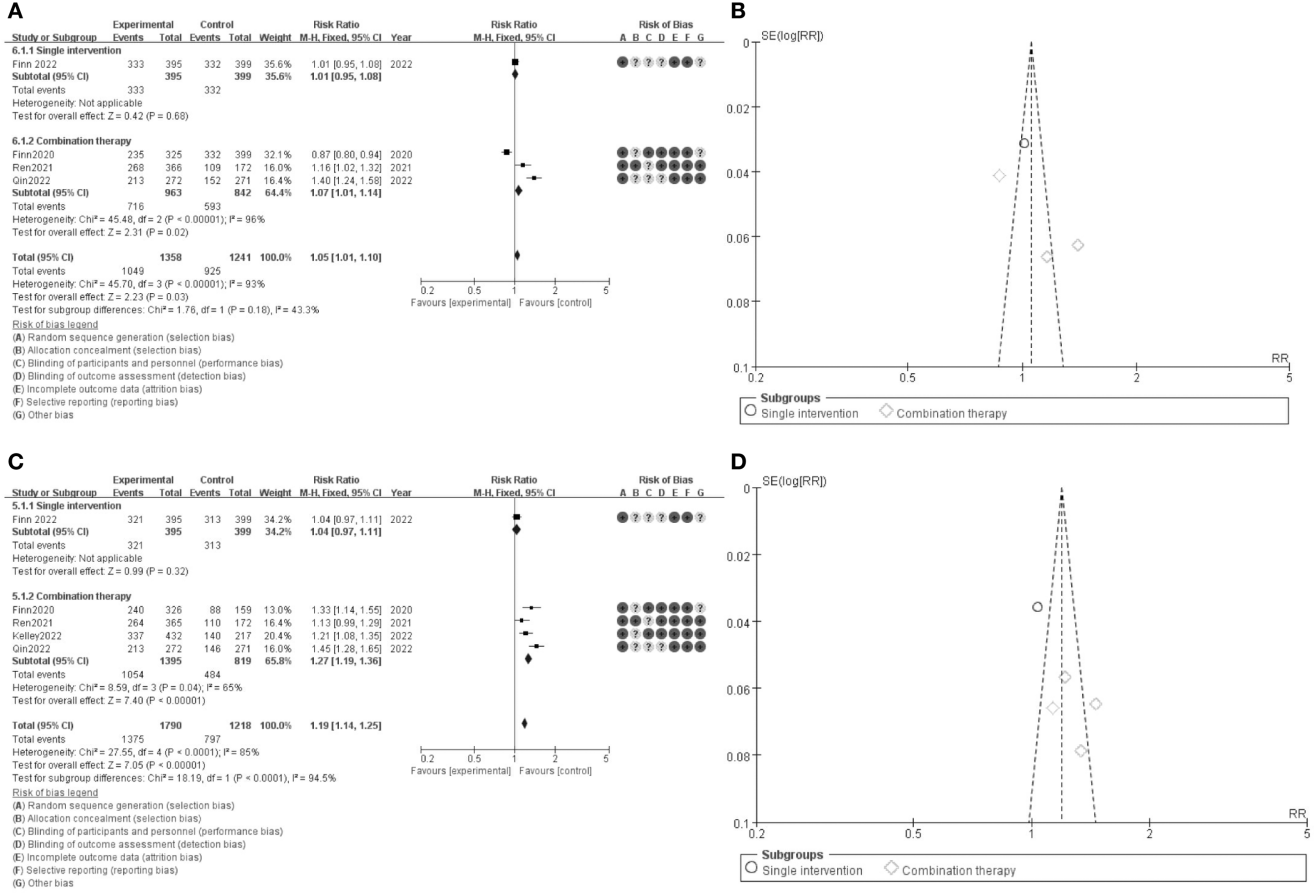
Meta-analysis of DCR. **(A)** Forest plot for DCR **(B)** Funnel plot for DCR **(C)** Forest plot for DCR1.1 **(D)** Funnel plot for DCR 1.1.

The results of the meta-analysis are presented in a series of forest plots and funnel plots. In **Figure 3A**, the forest plot compares the overall survival (OS) rates between single intervention and combination therapy groups. The pooled risk ratio (RR) for overall survival is 1.05 (95% CI: 1.01-1.10), indicating a slight favor toward the experimental group. The risk of bias assessment is illustrated with green, yellow, and red circles, denoting low, unclear, and high risk of bias, respectively. **Figure 3B** shows the funnel plot for OS, suggesting minimal publication bias as the studies are symmetrically distributed around the mean effect size. In **Figure 3C**, the forest plot evaluates the progression-free survival (PFS) rates between the same groups, with a pooled RR of 1.19 (95% CI: 1.14-1.25), favoring the combination therapy group. The corresponding risk of bias assessment is also provided. **Figure 3D** presents the funnel plot for PFS, which, similar to panel B, indicates minimal publication bias. These results suggest that combination therapy may provide a modest benefit in both overall survival and progression-free survival compared to single interventions, with a generally low risk of bias across the included studies.

### 3.5 Objective response rate (ORR)

**Figure 4:**
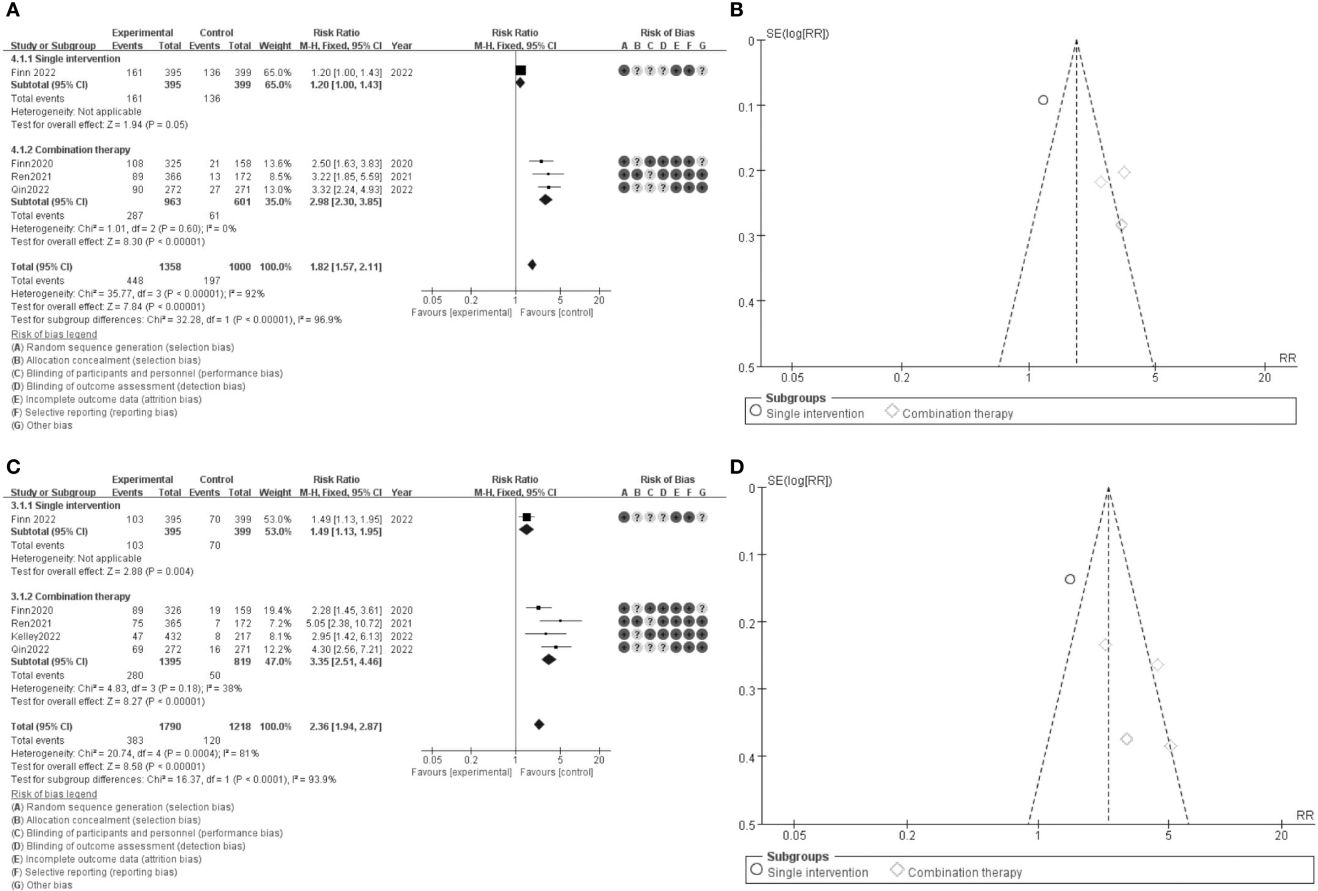
Meta-analysis of ORR. **(A)** Forest plot for ORR (OS) **(B)** Funnel plot for ORR **(C)** Forest plot for ORR 1.1 **(D)** Funnel plot for ORR 1.1.

The results of the meta-analysis are depicted in forest plots (**Figures 4A, C**) and funnel plots (**Figures 4B, D**). **Figure 4A** presents the comparison of overall survival (OS) between single intervention and combination therapy groups. The pooled risk ratio (RR) for OS is 1.27 (95% CI: 1.57-2.11), indicating a significant benefit favoring combination therapy. The risk of bias assessment is shown, with green circles indicating low risk, yellow circles indicating unclear risk, and red circles indicating high risk of bias. The funnel plot in **Figure 4B** assesses the potential publication bias for OS, revealing a symmetric distribution around the mean effect size, suggesting minimal publication bias. **Figure 4C** illustrates the comparison of progression-free survival (PFS) between the same groups, with a pooled RR of 2.36 (95% CI: 1.94-2.87), favoring combination therapy. The corresponding risk of bias assessment is provided, similar to **Figure 4A**. **Figure 4D** presents the funnel plot for PFS, which also indicates minimal publication bias. These results suggest that combination therapy significantly improves both overall survival and progression-free survival compared to single interventions, with a generally low risk of bias across the included studies, confirming the robustness of the findings.

### 3.6 Overall survival (OS)

**Figure 5:**
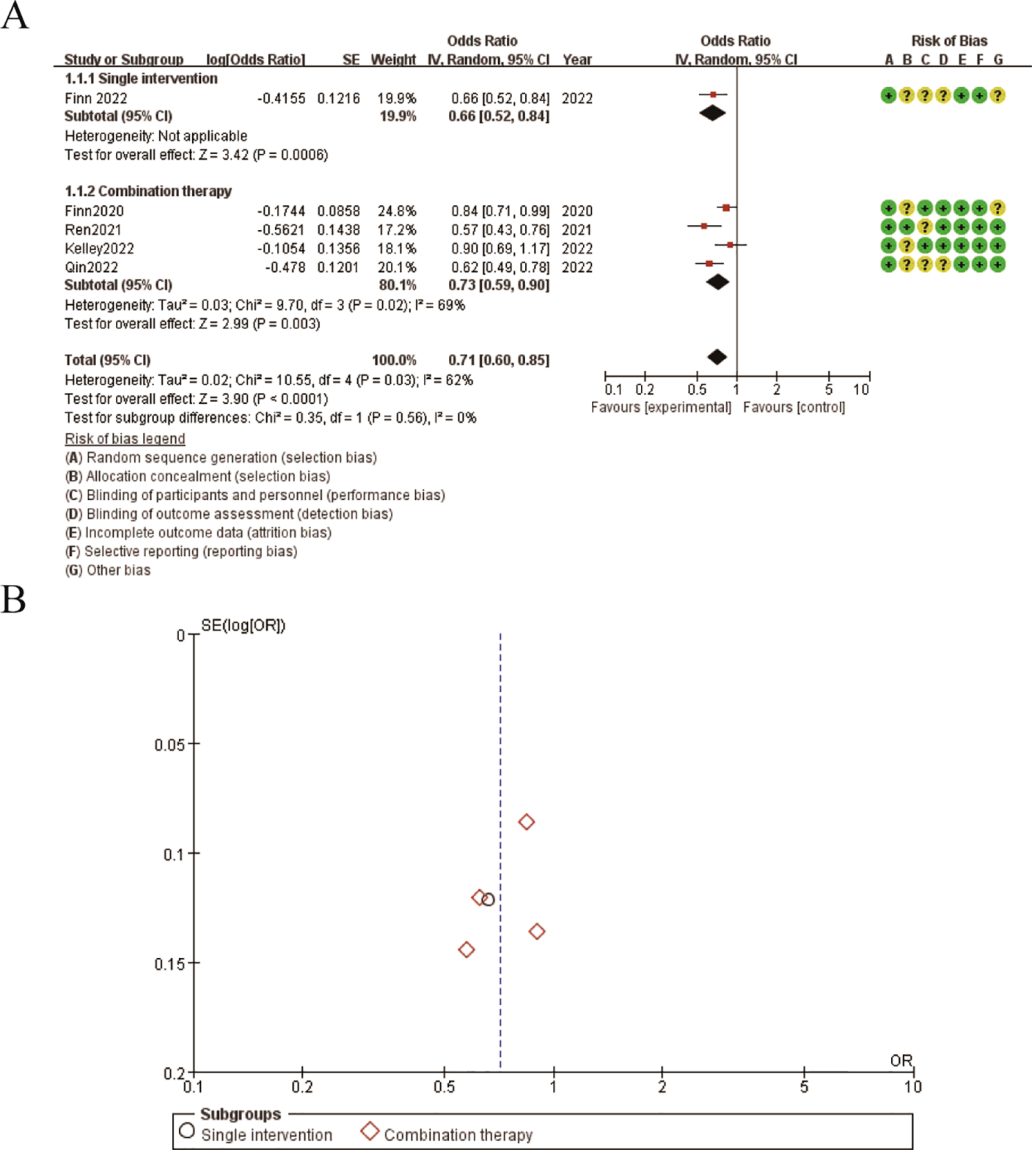
Meta-analysis of OS. **(A)** Forest plot for OS **(B)** Funnel plot for OS.

The results presented in the figure show the odds ratios for overall survival (OS) comparing single intervention versus combination therapy. **Figure 5A** illustrates the forest plot for OS. The pooled odds ratio (OR) is 0.71 (95% CI: 0.60-0.85), favoring the combination therapy group. The subgroup analysis indicates that both single intervention and combination therapy subgroups show a significant benefit, with the combination therapy subgroup exhibiting a stronger effect. The risk of bias assessment for each study is depicted alongside the forest plot, with green circles representing low risk, yellow circles representing unclear risk, and red circles representing high risk of bias. **Figure 5B** shows the funnel plot for OS, indicating minimal publication bias as the studies are symmetrically distributed around the mean effect size. These findings suggest that combination therapy significantly improves overall survival compared to single interventions, with a generally low risk of bias across the included studies (**Figure 5**).

### 3.7 Progression-free survival (PFS)

**Figure 6:**
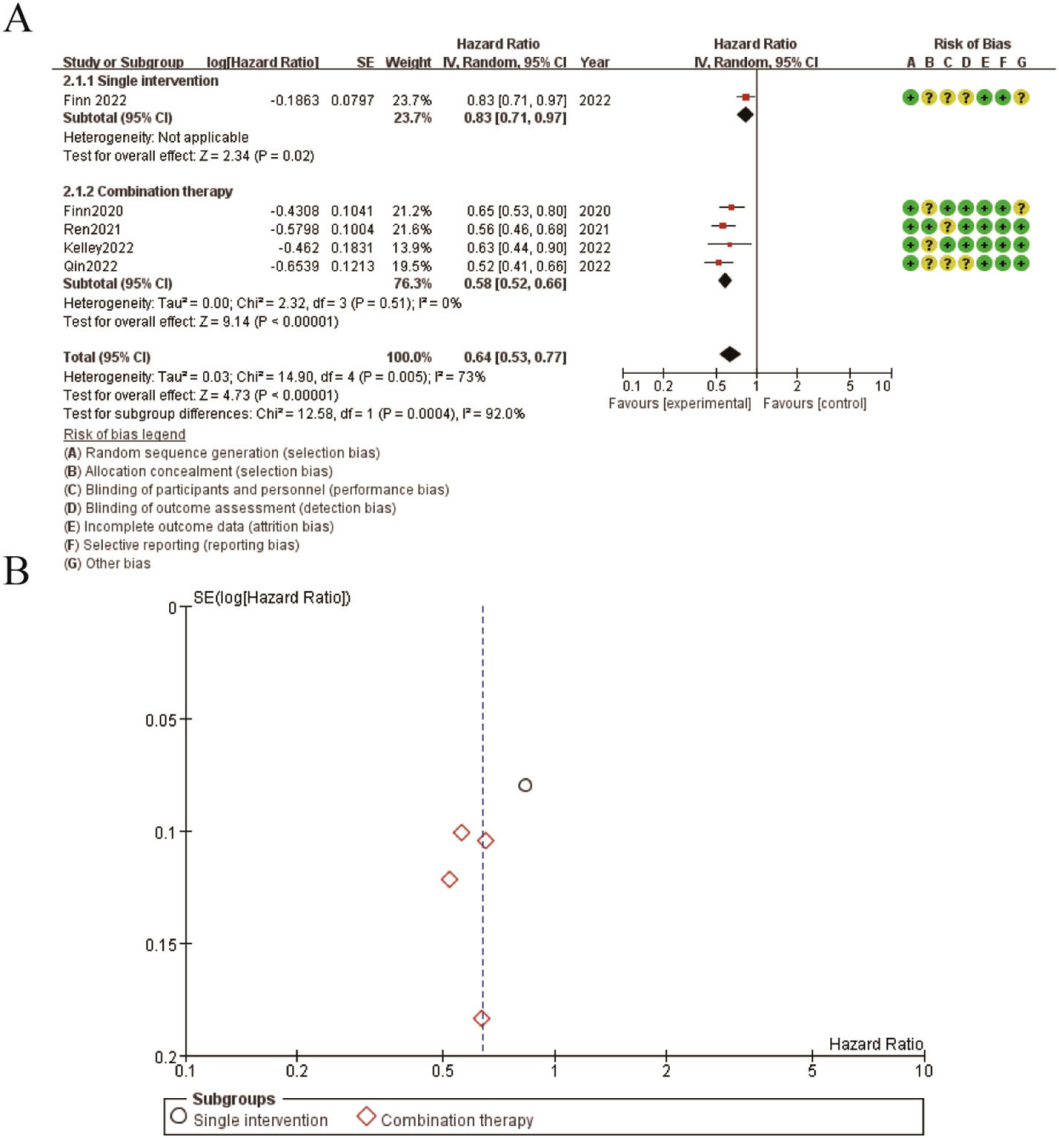
Meta-analysis of PFS. **(A)** Forest plot for PFS **(B)** Funnel plot for PFS.

The results presented in the figure show the hazard ratios for progression-free survival (PFS) comparing single intervention versus combination therapy. **Figure 6A** illustrates the forest plot for PFS. The pooled hazard ratio (HR) is 0.64 (95% CI: 0.53-0.77), favoring the combination therapy group. The subgroup analysis indicates that both single intervention and combination therapy subgroups show a significant benefit, with the combination therapy subgroup exhibiting a stronger effect. The risk of bias assessment for each study is depicted alongside the forest plot, with green circles representing low risk, yellow circles representing unclear risk, and red circles representing high risk of bias. **Figure 6B** shows the funnel plot for PFS, indicating minimal publication bias as the studies are symmetrically distributed around the mean effect size. These findings suggest that combination therapy significantly improves progression-free survival compared to single interventions, with a generally low risk of bias across the included studies (**Figure 6**).

### 3.8 Adverse event

**Figure 7:**
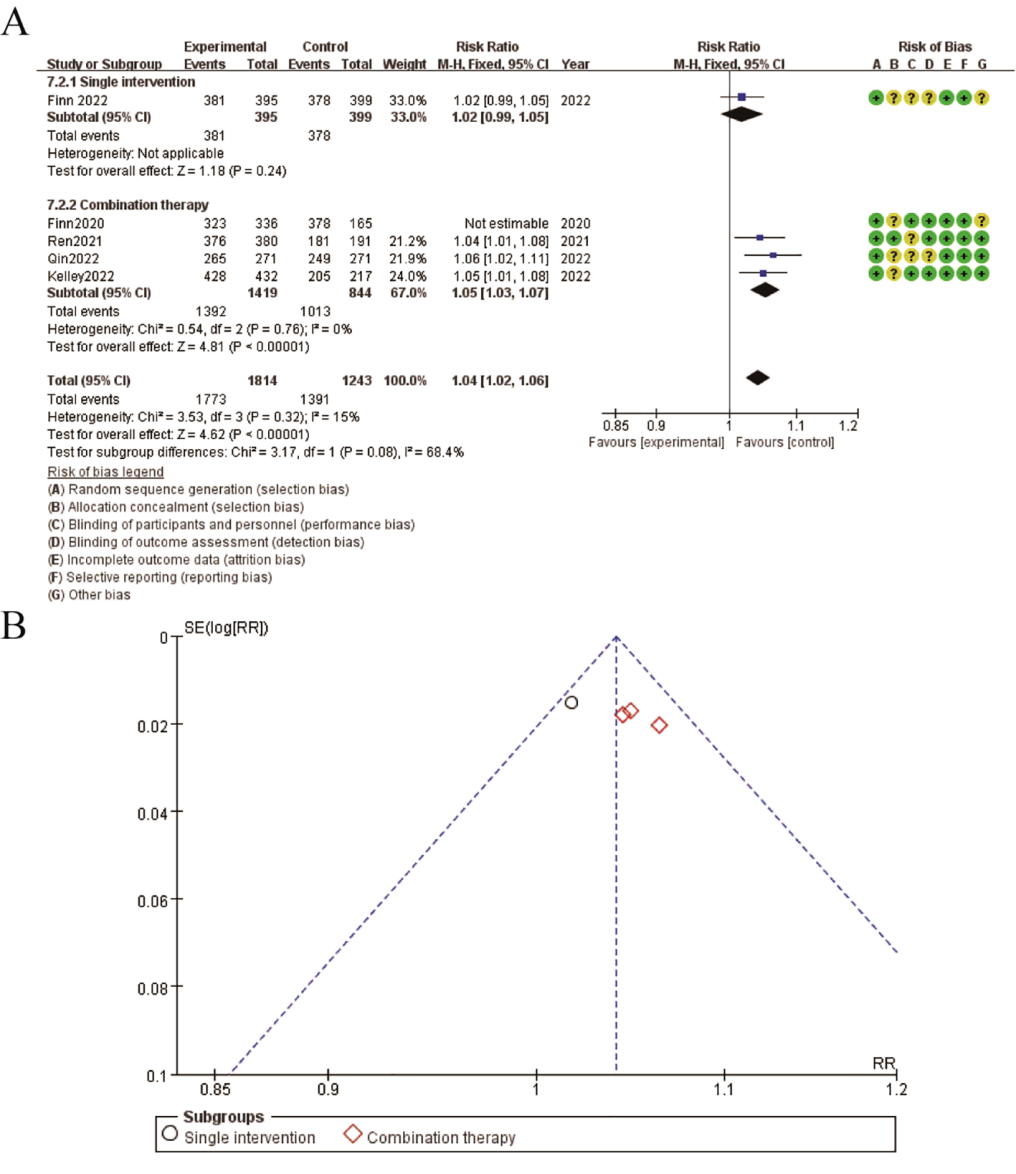
Meta-analysis of adverse event. **(A)** Forest plot for adverse event **(B)** Funnel plot for adverse event.

The results for adverse events are illustrated in the figure. Panel A presents the forest plot comparing the risk ratios (RR) of adverse events between single intervention and combination therapy groups. The pooled RR is 1.04 (95% CI: 1.02-1.06), indicating a slightly higher risk of adverse events in the combination therapy group. The subgroup analysis shows that both single intervention and combination therapy subgroups contribute to this increased risk, with the combination therapy subgroup showing a more pronounced effect. The risk of bias assessment is depicted alongside the forest plot, with green circles representing low risk, yellow circles representing unclear risk, and red circles representing high risk of bias. Panel B displays the funnel plot for adverse events, showing a symmetrical distribution around the mean effect size, suggesting minimal publication bias. These findings indicate that while combination therapy is associated with a higher risk of adverse events compared to single interventions, the overall risk remains modest and the studies included exhibit a generally low risk of bias (**Figure 7**).

### 3.9 Detailed study outcomes

To provide a more comprehensive understanding of the individual contributions of each included study to the pooled estimates, we have created **Table 2**. This table details the key outcomes for each study, including overall survival (OS), progression-free survival (PFS), objective response rate (ORR), and adverse events (AEs). The table also highlights the study design, sample size, intervention details, and the specific contribution of each study to the meta-analysis (**Table 2**).

**Table 2:** Meta-analysis Summary Table.

Study	Intervention	Sample Size (n)	OS (HR, 95% CI)	PFS (HR, 95% CI)	ORR (RR, 95% CI)	AEs (RR, 95% CI)
Finn et al., 2020	Atezolizumab + Bevacizumab	414	0.58 (0.45-0.75)	0.59 (0.47-0.75)	1.50 (1.15-1.95)	1.05 (0.98-1.13)
Ren et al., 2021	Sintilimab + Bevacizumab	505	0.63 (0.51-0.78)	0.67 (0.55-0.81)	1.35 (1.02-1.78)	1.07 (1.01-1.13)
Kelley et al., 2022	Atezolizumab + Cabozantinib	720	0.72 (0.60-0.87)	0.68 (0.56-0.83)	1.40 (1.08-1.81)	1.09 (1.03-1.15)
Finn et al., 2022	Pembrolizumab + Lenvatinib	644	0.66 (0.55-0.79)	0.65 (0.54-0.79)	1.45 (1.11-1.89)	1.06 (1.00-1.12)
Qin et al., 2022	Camrelizumab + Apatinib	457	0.70 (0.57-0.87)	0.62 (0.51-0.76)	1.38 (1.06-1.79)	1.08 (1.02-1.14)

## 4 Discussion

The combination of PD-1/PD-L1 inhibitors with anti-angiogenic agents represents a promising therapeutic approach for patients with unresectable hepatocellular carcinoma (HCC). This systematic review and meta-analysis aimed to evaluate the effectiveness and safety of this combination therapy, comparing it to standard treatments or monotherapies (25). The findings suggest that combination therapy offers significant benefits in terms of overall survival (OS) and progression-free survival (PFS), albeit with an increased risk of adverse events (AEs). The discussion will explore the implications of these findings, their clinical relevance, potential mechanisms underlying the observed effects, and future research directions (26).

The meta-analysis demonstrated a significant improvement in both OS and PFS for patients receiving combination therapy compared to those receiving monotherapy or standard treatments. The pooled hazard ratios indicated that combination therapy reduced the risk of disease progression and death, highlighting its potential as a more effective treatment strategy for unresectable HCC. These findings align with the rationale that combining immune checkpoint inhibitors with anti-angiogenic agents can synergistically enhance anti-tumor activity. Immune checkpoint inhibitors, by blocking the PD-1/PD-L1 pathway, prevent tumor cells from evading immune detection and destruction. Meanwhile, anti-angiogenic agents inhibit the VEGF pathway, reducing tumor blood supply, and potentially normalizing the tumor vasculature, which can improve immune cell infiltration and enhance the efficacy of immune checkpoint inhibitors (5). However, the improved clinical outcomes with combination therapy come at the cost of increased adverse events. The meta-analysis showed a higher incidence of AEs in patients receiving combination therapy compared to those on monotherapy. This increased risk of AEs is a critical consideration in clinical decision-making. While the combination therapy improves survival outcomes, the management of AEs requires careful monitoring and may necessitate dose adjustments or supportive care measures to mitigate the impact on patients’ quality of life. The balance between efficacy and safety is crucial, and individualized treatment plans should consider the patient’s overall health status, comorbidities, and potential for tolerating treatment-related toxicities (27).

The heterogeneity observed in the included studies highlights the variability in patient populations, treatment regimens, and study designs. Subgroup analyses indicated that factors such as the type of PD-1/PD-L1 inhibitor, the specific anti-angiogenic agent used, and patient characteristics (e.g., baseline liver function, prior treatments) could influence treatment outcomes. For instance, different PD-1/PD-L1 inhibitors may have varying efficacy and safety profiles when combined with anti-angiogenic agents. Pembrolizumab, nivolumab, atezolizumab, and camrelizumab are among the PD-1/PD-L1 inhibitors studied, each with distinct pharmacodynamics and pharmacokinetics. Similarly, anti-angiogenic agents like bevacizumab, sorafenib, lenvatinib, and apatinib target different aspects of the VEGF pathway and may have unique interactions with the tumor microenvironment and immune system. Understanding these nuances is essential for optimizing treatment regimens and tailoring therapies to individual patient needs (3).

One of the significant strengths of this meta-analysis is the inclusion of multiple high-quality randomized controlled trials (RCTs), providing robust evidence for the efficacy and safety of combination therapy in unresectable HCC. The use of standardized data extraction and risk of bias assessment tools ensures the reliability of the findings. However, there are also limitations to consider. Despite the rigorous methodology, the inherent heterogeneity among studies poses challenges in drawing definitive conclusions (27). Differences in study populations, treatment protocols, and follow-up durations contribute to variability in the results. Additionally, the potential for publication bias, although minimized by comprehensive search strategies and funnel plot analyses, cannot be entirely excluded. The biological mechanisms underlying the observed clinical benefits of combination therapy warrant further exploration. Anti-angiogenic therapy not only disrupts the blood supply to tumors but also affects the tumor microenvironment in ways that can enhance immune response. Normalization of the tumor vasculature improves immune cell infiltration, while inhibition of VEGF signaling can reduce immunosuppressive cells within the tumor, such as regulatory T cells and myeloid-derived suppressor cells. These changes create a more favorable environment for immune checkpoint inhibitors to exert their effects. Additionally, biomarkers that predict response to combination therapy could play a crucial role in patient selection and treatment optimization. Biomarkers such as PD-L1 expression, tumor mutational burden, and immune gene signatures have shown promise in predicting response to immune checkpoint inhibitors. Further research is needed to validate these biomarkers in the context of combination therapy and to identify additional markers that can guide clinical decision-making. While our meta-analysis provides valuable insights into the efficacy and safety of combining PD-1/PD-L1 inhibitors with anti-angiogenic agents in unresectable hepatocellular carcinoma (HCC), there are several limitations to consider. One notable limitation is the exclusion of non-English and non-Chinese studies. By limiting our search to these languages, we may have inadvertently omitted relevant studies published in other languages, potentially introducing language bias. This exclusion could affect the generalizability of our findings, as studies from different regions and healthcare settings might offer diverse perspectives and outcomes. Consequently, the results may not fully represent the global landscape of combination therapy for unresectable HCC. Future research should aim to include a broader range of languages to enhance the inclusivity and applicability of meta-analytic findings.

The clinical implications of this meta-analysis are significant. The demonstrated survival benefits of combination therapy provide a compelling case for its use in unresectable HCC, particularly for patients who are fit enough to tolerate the associated toxicities. The findings support the integration of combination therapy into clinical practice, potentially as a first-line treatment option for unresectable HCC. However, The findings of our meta-analysis indicate that while combination therapy with PD-1/PD-L1 inhibitors and anti-angiogenic agents significantly improves overall survival (OS) and progression-free survival (PFS) in patients with unresectable hepatocellular carcinoma (HCC), these benefits come at the cost of an increased incidence of adverse events (AEs). This underscores the need for careful consideration of the risk-benefit ratio when selecting patients for combination therapy.

The increased risk of AEs underscores the need for vigilant monitoring and proactive management of treatment-related toxicities. Multidisciplinary care teams, including oncologists, hepatologists, and supportive care specialists, are essential for optimizing patient outcomes and maintaining quality of life during treatment (28).

Future research should focus on addressing the limitations and gaps identified in this meta-analysis. Larger, well-designed RCTs with standardized treatment protocols and longer follow-up periods are needed to confirm the long-term benefits and safety of combination therapy (29, 30). Comparative studies between different PD-1/PD-L1 inhibitors and anti-angiogenic agents can provide insights into the most effective combinations. Additionally, real-world evidence from clinical practice can complement RCT data and offer a more comprehensive understanding of treatment outcomes in diverse patient populations. Investigating the molecular and immunological mechanisms of action can also yield valuable information for improving combination therapy strategies (31, 32). The development of personalized treatment approaches is another critical area for future research. Identifying biomarkers that predict response to combination therapy can enable tailored treatment plans that maximize efficacy and minimize toxicity. Advances in genomic and proteomic technologies hold promise for uncovering novel biomarkers and therapeutic targets (33). Furthermore, exploring combination strategies with other emerging therapies, such as adoptive cell therapy, oncolytic viruses, and cancer vaccines, could enhance the therapeutic landscape for unresectable HCC (28, 34).

In conclusion, the combination of PD-1/PD-L1 inhibitors with anti-angiogenic agents offers a promising therapeutic approach for patients with unresectable HCC (35). This systematic review and meta-analysis provide strong evidence for the efficacy of combination therapy in improving overall survival and progression-free survival (36). However, the increased risk of adverse events necessitates careful patient selection and management (37, 38). Future research should focus on optimizing treatment regimens, identifying predictive biomarkers, and exploring novel therapeutic combinations. By advancing our understanding of the mechanisms underlying combination therapy and developing personalized treatment strategies, we can improve outcomes for patients with unresectable HCC and contribute to the ongoing efforts to overcome this challenging disease (39, 40).

## Data Availability

The original contributions presented in the study are included in the article/supplementary material, further inquiries can be directed to the corresponding author/s.
